# Water, women and disability: Using mixed-methods to support inclusive WASH programme design in Vanuatu

**DOI:** 10.1016/j.lanwpc.2021.100109

**Published:** 2021-03-02

**Authors:** Islay Mactaggart, Sally Baker, Luke Bambery, Judith Iakavai, Min Jung Kim, Chloe Morrison, Relvie Poilapa, Jeanine Shem, Phillip Sheppard, Jamie Tanguay, Jane Wilbur

**Affiliations:** a International Centre for Evidence in Disability (ICED), London School of Hygiene & Tropical Medicine, Keppel Street, London WC1E 7HT, UK; b Melbourne School of Population Health, University of Melbourne, 207 Bouverie St, Carlton, VIC 3053, Australia; c World Vision Vanuatu, Rue Artoi M/S, P.O Box 247, Port Vila, Vanuatu; d Vanuatu Society for People with Disability (VSPD), Vanuatu; e Vanuatu National Statistics Office, Port Vila, Vanuatu

**Keywords:** Disability, Gender, WASH

## Abstract

**Background:**

Adequate access to water, sanitation and hygiene (WASH) is imperative for health and wellbeing, yet people with disabilities, people with incontinence and people who menstruate often experience unmet WASH requirements.

**Methods:**

In 2019 we completed a mixed-methods study in two provinces of Vanuatu, (SANMA and TORBA). The study comprised 1) a population-based disability survey using the Washington Group Short-Set 2) a nested case-control study to explore associations between WASH, disability and gender, and 3) an in-depth qualitative assessment of the experiences of WASH users with additional requirements: people with and without disabilities who menstruate, or experience incontinence.

**Finding:**

11,446 households (response rate 85%) were enrolled into the survey. All-age disability prevalence across the two provinces was 2.6% (95% Confidence Interval 2.5–2.8), increasing with age. 814 people with, and 702 people without disabilities participated in the case-control study. People with disabilities were statistically more likely to experience barriers in seven of eight intra-household indicators. WASH-related stigma, reliance on informal caregivers, and under-resourcing of WASH personnel were critical issues for people who menstruate or experience incontinence.

**Interpretation:**

People with disabilities, people with incontinence and people who menstruate in Northern Vanuatu face continued challenges in accessing safe, affordable and appropriate WASH that meets their requirements. Outputs from this study have supported progression towards gender and disability-inclusive WASH programming in the area and highlighted the value of mixed-methods research.

**Funding:**

The research was funded by the Australian Government's Water for Women fund and donations from the Australian public.

**Research in Context Panel**

Evidence before this study: Safely managed drinking water, sanitation and hygiene (WASH) supports general health, prevents disease and enables participation in major life areas such as education and livelihoods. Pervasive inequalities in access to WASH between countries, communities and households is well documented. A smaller evidence base across a number of low- and middle-income countries has identified additional barriers in accessing WASH facilities experienced by people with disabilities, particularly within their homes. People with and without disabilities who experience incontinence and/or menstruate may have additional unmet WASH requirements, but few data on this are available globally.

Combined coverage of basic water and sanitation (using UNICEF/WHO Joint Monitoring Programme definitions) in Vanuatu is amongst the lowest in the world, with previous evidence suggesting that less than half of population have access to safely managed drinking water, less than two thirds have access to at least basic sanitation and less than three quarters have a basic handwashing facility at home. Data are urgently needed on the situation of people with additional WASH requirements or who experience additional barriers to WASH in Vanuatu, to support the development of inclusive WASH programmes and policies.

*Added value of this study:* This comprehensive mixed-methods study across SANMA and TORBA, the two northern-most provinces of Vanuatu, was a collaboration between WASH and disability actors in Vanuatu, and researchers focused on building the evidence base on disability and WASH in Global Health. The study estimated the prevalence of disability using the Washington Group Short Set, allowing comparability with other settings, and established a client register of over 11,000 households for the programme team. The quantitative component provided data on the scope of inequalities experienced by different groups, and identified sub-groups most at risk. The qualitative component provided rich contextualisation of the study findings, to drive evidence-based programme design.

*Implications of all the available evidence:* People with disabilities, people with incontinence and people who menstruate in northern Vanuatu lack adequate support and resources to meet their WASH requirements. Additional policy and programme resourcing is required to close this gap, particularly in the context of the COVID-19 pandemic and the integral role of WASH in mitigating infection risk to vulnerable groups.

Alt-text: Unlabelled box

## 1 Introduction

Globally, people with disabilities often experience substantial barriers to accessing water, sanitation and hygiene (WASH), particularly within their households [1,2]. Barriers are wide-ranging and variable, but may include the physical inaccessibility of WASH facilities or the path towards them, risk of violence or stigma, inaccessible WASH information, unavailability of support and lack of involvement in policy and practice [3], [4], [5]. Internationally, WASH access tends to be substantially lower for households in rural locations [6,7].

Among people with disabilities, a limited evidence base suggests that older people, people with more significant activity limitations and those with mobility or self-care limitations are most likely to face WASH barriers [[1], [2], [3],^8^,^9^]. In addition, people who experience incontinence, and women and girls who menstruate (see Table 1 for definitions of key terms), may have further unmet requirements for water and adequate WASH facilities [10], [11], [12].

**Table 1:** Tools and definitions.

Age Group (years)	Tool	Domains	Threshold
Disability Tools and definitions			
0–4	*Excluded from disability screening*		
5–17 (proxy report)	Washington Group (WG) Short Set (WGSS) [21]	Seeing, hearing, walking, self-care, understanding/ being understood, remembering/ concentrating	Any domain “a lot of difficulty” or “cannot do”
18+	WGSS [21]	As above	As above
18+	WG Mental Health Questions [21]	Four additional questions on anxiety and depression symptom frequency and severity. Note, anxiety and depression estimates are not included in the reported prevalence estimates	Reporting experiencing symptoms “every day” and “a lot”
Wellbeing Tool and Definition			
16+	Cantril Ladder for Subjective Wellbeing [22,23]	Participants are asked to imagine a ladder with 11 rungs 0–10, where 0 represents “Not at all satisfied” and 10 represents “Completely satisfied”. Participants report overall, how satisfied they are with their life as a whole presently, and how satisfied with their life they expect to be in 5 years’ time	Thriving: ≥7 present, and ≥8 in the future Struggling: Neither thriving nor suffering Suffering: ≤4 present and ≤4 in the future

Category	Sub Category	Definition	

Drinking Water Definitions [24]			
Improved	Safely managed	Drinking water from an improved water source (one that protects from outside contamination, in particular from faecal matter) which is located on premises, available when needed and free from faecal and priority chemical contamination	
	Basic	Drinking water from an improved source, provided collection time is not more than 30 minutes for a roundtrip including queuing	
	Limited	Drinking water from an improved source for which collection time exceeds 30 minutes for a roundtrip including queuing	
Unimproved	Unimproved	Drinking water from an unprotected dug well or unprotected spring	
	Surface water	Drinking water directly from a river, dam, lake, pond, stream, canal or irrigation canal	
Sanitation Definitions [24]			
Improved	Safely managed	Use of improved facilities which are not shared with other households and where excreta are safely disposed in situ or transported and treated off-site	
	Basic	Use of improved facilities which are not shared with other households	
	Limited	Use of improved facilities shared between two or more households	
Unimproved	Unimproved	Use of pit latrines without a slab or platform, hanging latrines or bucket latrines	
	Open defecation	Disposal of human faeces in fields, forests, bushes, open bodies of water, beaches and other open spaces or with solid waste	

Menstrual Hygiene Management (MHM) Definition		Women and adolescent girls using clean menstrual management material to absorb or collect blood, that can be changed in privacy as often as necessary for the duration of the menstruation period, using soap and water for washing the body as required, and having access to facilities to dispose of used menstrual management materials. They understand the basic facts linked to the menstrual cycle and how to manage it with dignity and without discomfort or fear [25].	
Incontinence Definition		Incontinence can be classified as faecal, urinary, or both. Urinary incontinence is defined as the involuntary loss of urine that is objectively demonstrable and is a social or hygienic problem [26,27]. Faecal, or bowel, incontinence is an inability to control bowel movements, resulting in the involuntary passage of stools [26].	

The ongoing COVID-19 pandemic has thrust the significance of adequate access to WASH–in particular hygiene, historically overlooked by international development actors and governments alike–into the spotlight [13]. Specifically, frequent hand washing with soap and water has been a pillar of global health messaging to prevent the spread of the virus [14]. In parallel, a growing literature suggests that people with underlying health conditions, which includes many people with disabilities and older adults, are at increased risk of severe negative health outcomes from COVID-19, actively requiring inclusive COVID-19 prevention strategies to mitigate these risks [15], [16], [17].

In 2019 we completed the Water, Women and Disability Study, a mixed-methods study undertaken in the two northern-most provinces of Vanuatu, an island nation in the South Pacific with a total national population of under 300,000 people [18,19]. The study provided the baseline assessment to inform the development of the Laetem Dak Kona (LDK) project, an inclusive WASH programme targeting women and people with disabilities across the two provinces [20].

This manuscript describes how mixed methods were used to generate evidence for inclusive WASH policy and programming in Vanuatu.

## 2 Methods

### 2.1 Study setting and design

The study was completed in TORBA and SANMA provinces. The terrain across the provinces is predominantly small to medium-sized islands. There is one urban location, Luganville, at the Southern tip of the largest island, Espritu Santo, which is the residence of 20% of the population of the two provinces [28]. The population is predominantly ni-Vanuatu, a collective term used to describe various smaller Melanesian ethnic groups across the islands [29,30]. Travel between islands is mostly by commercial or charter plane, or boat. The three official languages are English, French and Bislama (a variation of Melanesian Pidgin), but local vernacular languages are used by the majority of households in both provinces [29,31]. Previously described challenges in implementing successful WASH programmes in Pacific Island States include implementers’ lack of contextual knowledge, the remoteness of rural island settlements and lack of participation of marginalised groups in programme design [32].

We used a mixed methods approach comprised of both qualitative and quantitative components. The quantitative component included 1) a comprehensive population-based survey to establish disability prevalence and develop a programme client register for LDK 2) a nested case-control study to explore associations between WASH, disability and gender. The qualitative component focused on an in-depth qualitative assessment of the experiences of WASH users with additional requirements: women and girls who menstruate, and people who experience incontinence (with and without disabilities). This comprised structured observations, a market survey of menstrual and incontinence materials, in-depth interviews (IDIs) and PhotoVoice. The market survey involved buying a selection of menstrual hygiene and incontinence products from local shops, showing them to participants during the interview and discussing if they had ever seen or used them before, their preference and reasons for this. Participants also ranked the products according to preference.

Since we completed data collection, Vanuatu has experienced a powerful tropical storm (Cyclone Harold, in April 2020), which caused major damage to Luganville and surrounding areas, disrupting water and destroying food supplies [33]. At the time of press, the country remains in an extended State of Emergency in response to both the cyclone and the COVID-19 pandemic, but has not recorded any cases of transmission in the community [34].

### 2.2 Quantitative component

#### 2.2.1 Household listing and disability prevalence survey

A complete household listing (all ages) of the total population was undertaken across an expected 14,000 households in TORBA and SANMA provinces between March and July 2019. This was inclusive of approximately 8000 evacuees from the island of Ambae in the neighbouring Penama Province (following volcanic activity in 2017–2018), expected to have settled permanently in Luganville [35].

Ten teams of interviewers were recruited from across the study area and received two weeks training (58 interviewers in total). Alongside gender parity, we sought meaningful inclusion of persons with disabilities in the survey teams, through active recruitment of persons with disabilities via local Organisations of People with Disabilities (OPDs), civil society and vocational training programmes. The structured training programme was developed and delivered collaboratively between study partners, comprising sessions on study background, methods, protocol and ethics, field practice and pilot testing, and safeguarding.

2016 Mini Census data and Enumeration Area (EA) maps were acquired from the Vanuatu National Statistics Office. All households that had either lived in the study area for at least 12 months or, if not, intended to remain there for a minimum of 12 months following data collection, were eligible for enrolment. Each team completed all data collection in an EA in one to two days before moving to the next. If a dwelling within an EA was found to be inhabited but unattended, up to two repeat visits were undertaken. If the whole household was unavailable following this, the household was recorded as unavailable.

For each household that agreed to participate in the study, a household roster was first completed. Each household member aged 5+ was then screened for reported functional limitations using the Washington Group tools as described in Table 1. Adults aged 18+ self-reported, and adult caregivers reported for all children 5–17. If adults (or adult caregivers of children 5–17) were not available on the day of data collection, up to two repeat visits were made as feasible. If repeat visits were not feasible, or two unsuccessful repeat visits had already been made, an adult household member acted as proxy for the unavailable adult/adult caregiver, which was recorded on the data entry form.

#### 2.2.2 Nested case-control study

The nested case-control study recruited a sub-sample of survey participants identified as having a disability age 5+ (“cases”) and an equal number of people without disabilities (“controls”). Controls were matched to cases by EA, sex and age. For children, the control needed to be a child within one year of age (+/-) of the child case. For adults, the control needed to be an adult within three years of age (+/-) of the adult control.

Based on expected disability prevalence and expected differences in sanitation scores for people with and without disabilities [8], a sample size of 800 people with disabilities, matched by age-sex group and community to 800 people without disabilities was determined to be sufficient to provide adequate power to assess differences in WASH access and experience. The sample was stratified to ensure adequate representation of young people with disabilities. In addition to in-depth structured modules on household and intra-household WASH, menstruation and incontinence, the case-control questionnaire included modules on education, livelihoods, health, social participation and wellbeing.

### 2.3 Qualitative component

A participatory framework underpinned the qualitative research methodology, ensuring local relevance, ownership and input of local expertise. The qualitative component of the study further explored the situation for people with additional WASH requirements, complementing the quantitative component focus on the scope of unmet needs.

#### 2.3.1 Study population and sample size

The study population and inclusion criteria were:

• 19 women and men with a disability, 18 to 65+ years, who experience incontinence at least two times a week.
• Eight women and men without a disability, 18 to 65+ years, who experience incontinence at least two times a week.
• Nine women with a disability, aged 18–45 years, who menstruate regularly.
• Eight women without a disability, aged 18–45 years, who menstruate regularly.
• Five national level policy makers.
• Four implementers of health and / or WASH interventions.
• Seven individuals working for disability service providers and rights organisations.

Purposeful sampling was applied to select participants who met the inclusion criteria from the nested case-control study. Individuals with and without a disability were asked the Washington Group (WG) Short Set questions (WGSS) directly to confirm their disability status [36], and their age. They were also asked if they experience urinary or faecal incontinence at least two times a week and/or if they menstruated. Participants who did not meet the inclusion criteria were excluded.

We intentionally sampled women and men with a range of functional limitations, ages and locations and matched them with women and men without disabilities of similar ages and locations so that we could compare experiences. Some participants did not meet the inclusion criteria, so we applied snowball sampling (a method whereby participants identify other people to interview) to ensure representation across the different variables. If participants did not fully understand the consent process, their caregivers were interviewed as proxy. 44 people with and without disabilities formed the sample size. Seventeen policy makers and implementers were purposively sampled through World Vision Vanuatu and the Vanuatu Society for People with Disability (VSPD)'s networks, and snowball sampling was applied by the research team.

#### 2.3.2 Data collection methods and activities

To allow for methods triangulation, five different qualitative data collection methods were applied: IDIs, observation, Focus Group Discussions, Key Informant Interviews, and PhotoVoice and ranking. A description of each method, its purpose and the characteristics of the sample for each is detailed in Table 2. Note that the same participants were involved in IDIs (including the market survey and ranking) and observation.

**Table 2:** Qualitative methods and sample characteristics.

Method	Purpose	Description	Sample Characteristics
In-depth interview (IDI)	To understand access and barriers to accessing WASH facilities; experiences of ***incontinence*** and strategies applied to manage it	Interviews lasted between 40 minutes and 1.5 hours and were conducted in the participant's home. With consent, interviews carried out in Bislama and translated into English if JW (who does not speak Bislama) was present, and recorded on a voice recorder. Field notes were written after the interviews. If the participant did not fully understand the consent process, a proxy (caregiver) was interviewed instead.	The sample was drawn from rural and urban areas and comprised:People with disabilities: • 7 women who menstruate 18-45 (3 were interviewed by proxy) • 16 women and men experiencing incontinence 18-65 (includes 9 proxy in-depth interviews) People without disabilities: • 8 women and men experiencing incontinence 18-65
	To understand access and barriers to accessing WASH and MHM facilities; experiences of ***menstruation*** and strategies applied to manage it		
	To understand the ***menstrual*** materials available, used, and participant preference	Market survey, assessment of product and user preference with ranking: a selection of menstrual and incontinence management products bought in local shops were shown to participants during the interview. Researchers asked participants if they had ever seen or used the products, their preference and the rationale. They were also asked to rank them in order of least to most preferred product. A photo was taken of the order.	
	To understand the ***incontinence*** materials available, used, and participant preference		
Observation	To observe whether participants face any challenges using water, sanitation or bathing shelters (revised version of WEDC, WaterAid (2013) Accessibility and safety audit) [37]	After the interview, researchers watched participants demonstrate where they collected water, bathed and went to the toilet. Issues explored: distance from the home to the facility, accessibility of the route to the facility and the infrastructure; privacy, safety and security. Field notes were taken after the observation.	
Focus Group discussion	To explore access and barriers to accessing WASH and MHM facilities; experiences of ***menstruation*** and how it is managed	Discussions lasted between 1 and 2 hours, and were conducted in a community hall, a World Vision meeting room and via Skype. With consent, the discussions carried out in English, or Bislama and translated into English for JW, and recorded on a voice recorder. Field notes were written after the interviews.	8 women drawn from rural and urban areas without a disability, aged 18-45 years, who menstruate regularly
	To explore health and WASH related issues facing people with disabilities , disability services available and the challenges related to delivering those		7 representatives from disability service providers and Organisations of Persons with Disabilities
	To explore how public health policy priorities are identified, how policies are developed and implemented, and the space for civil society to participate within these mechanisms; levels of understanding of disability, MHM and incontinence, and commitment to disability inclusive WASH		4 professionals working in the area of Health
PhotoVoice*	To enable participants to represent their experiences related to ***incontinence*** visually and rank these according to perceived level of importance	Camera phones were lent to participants, who were asked to take five photos of things that made them feel happy, and five photos of their experiences related to managing menstruation and/or incontinence. Photos taken were shown to participants on a laptop, who were then interviewed about what issue they conveyed in each image. Participants provided a caption for each photo and ranked them according to level of importance. The process took 0.5 to 1 day per participant. All participants requested that their real names be credited whenever their photos and captions are used.	3 women and men with a disability, 18 to 65+ years who experience incontinence (1 conducted through a proxy)
	To enable participants to represent their experiences related to ***MHM*** visually and rank these according to perceived level of importance		2 women with a disability who menstruate, aged 18-45 years
Key informant interviews	To explore how WASH policy priorities are identified, how policies are developed and implemented, and the space for civil society to participate within these mechanisms; levels of understanding of disability, MHM and incontinence, and commitment to disability inclusive WASH	Key informant interviews conducted with policy makers and implementers were carried out face to face in participants’ offices, and lasted between 45 minutes and 1.5 hours. With consent, the discussions carried out in English, or Bislama, and recorded on a voice recorder. Field notes were written after the interviews.	1 professional working in the area of WASH
	To investigate knowledge of disability, incontinence and menstruation, training and resources provided on these topics; services provided to people with and without disabilities, and caregivers, and provision of incontinence and / or menstrual materials		1 healthcare professional, working in a rural location 1 healthcare professional, working in an urban location
	To explore WASH service implementation, knowledge of the issues faced by people with and without disabilities, how to address them, and challenges related to doing that		2 professionals working in the WASH sector

⁎ PhotoVoice is a visual research methodology, in which participants are loaned a camera, shown how to take photos and asked to take photos that represent their experiences related to the study issues [38]. The methodology has been used to explore WASH issues, including MHM and incontinence, in Nepal [39], Kenya [40], Malawi [5], Pakistan [41] and Ghana [42].

#### 2.3.3 Research team training

A female and male field researcher, a female research coordinator and a female World Vision programme manager participated in a one-week training led by the study team. Sessions covered the research design, how to administer data collection tools, how to conduct qualitative research ethically with people who have a disability and their caregivers, and how to discuss sensitive topics confidently and within a culturally appropriate context. Data collection tools were tested and revised during this week. The research team was coached throughout the data collection process.

### 2.4 Data management and analysis

Both quantitative research components used mobile data entry, facilitated through a partnership with Digicel Vanuatu to improve mobile network coverage. The Open Data Kit [43] was used to build a mobile version of the questionnaires with inbuilt skip logic. Each interviewer was provided with a password-protected Android tablet and data SIM card. To ensure data protection and integrity, all data were encrypted on the tablet before being transferred via cellular data to a secure, encrypted server held at the London School of Hygiene & Tropical Medicine (LSHTM).

Quantitative data were analysed in STATA 14.0. Prevalence estimates were generated using the proportion command. Adjusted odds ratios were generated to compare outcomes between and within groups using a combination of binary and multinomial logistic regression to predict dependence where outcome variables were binary or categorical, respectively. All household-level regression models were multivariable to adjust for socio-economic status as a potential confounder, while individual-level models also included age, location and sex. Outcome variables are listed in each table, with independent variables specified in column headers and table footnotes. Definitions of disability and WASH outcomes are provided in Table 1. Self-reported sex was used as a proxy for gender, given that separate terminology for the latter does not exist in Bislama.

Wellbeing was measured using the Cantril Ladder for Subjective Wellbeing [22] and the Alternative Indicators for Wellbeing in Melanesia [44], results from the latter of which will be reported in a separate publication in this series. The Cantril Self-Anchoring Scale was used to categorise participants as “thriving”, “struggling” or “suffering” on a scale based on their composite Cantril Ladder scores [23].

A principal component analysis (PCA) was completed for case-control participants to establish household socioeconomic status (SES) quartiles, based on analysis of household assets, household head attributes, access to indigenous land and household characteristics.

Qualitative data was analysed iteratively: the research team met every day to discuss: 1) the day's interviews and the field notes to identify emerging themes, such as the challenges that inaccessible toilets presented to people with mobility limitations who experience incontinence, 2) interview techniques, any challenges faced when discussing the sensitive topics and how to manage that going forward, 3) the sample size to ensure we were reaching representation across each variable (e.g. women and men with and without disabilities who experience incontinence).

Voice recordings of the interviews were translated, transcribed into English and checked by the ni-Vanuatu research team members for accuracy. Any discrepancies were corrected before finalisation.

When data collection ended, the research team had a daylong meeting to group the findings into themes, discuss and analyse any connections between them. A thematic analytical approach was taken across IDIs, interviews from PhotoVoice, focus group discussions and key informant interviews to generate initial codes using Nvivo 11, search for themes, review and name the themes before writing a report. Discussions from the research team workshop fed into analyses.

Triangulation between quantitative and qualitative results was achieved through collaborative interpretation by the respective research leads (IZM and JW) and relevant national and international stakeholders. Quantitative and qualitative results per thematic area were presented together at participatory workshops, and compiled into a comprehensive Study Report following the same thematic approach. Structuring the findings by theme enables interpretation of the proportion of the population affected and scope of unmet needs from the quantitative components, together with rich contextualisation from the qualitative components, to drive evidence-based programme design.

### 2.5 Ethics

Ethical approval was gained from LSHTM's Observational Ethics Committee (Ref 16202/2019) and, in the absence of an Ethics Committee in Vanuatu, endorsement was provided in writing from the Ministry of Justice and Community Services.

Written informed consent was taken at three different stages of the quantitative data collection: 1) Household Listing–Household Head/Adult Key Informant; 2) Disability Screening–Individual disability screening for adults 18+ and proxy screening for children (5–17 years) and 3) Nested Case Control Study–Individual for adults 18+ and proxy for children (as above). At each point, an information sheet was read out or shared by the interviewer, describing the study purpose, procedures, benefits and risks, confidentiality of responses, and the eligible participant's right to refuse or withdraw at any time. Participants were given the option to provide their anonymised data for analytical purposes only, or for their contact details to be shared with project partners for programmatic purposes.

### 2.6 Role of the funding source

The LDK project is supported by the Australian Government's Water for Women fund and donations from the Australian public. The funding source did not play any role in the design or implementation of the research study, or the study write up.

## 3 Results

Of 13,500 households approached, 11,446 (response rate 85%) were enrolled into the disability prevalence survey. A total of 56,402 individuals were enumerated (average household size 4.9). The prevalence of disability in the population 5 years and older, excluding those who were unavailable or refused to respond, was 2.6% (95% Confidence Interval, 2.5–2.8) [Table 3]. Prevalence was higher in Luganville (3.5%, 3.2–3.8) than the rural settings, and positively associated with ageing. There was no significant difference by sex.

**Table 3:** Disability prevalence (Washington group short set standard definition).

	All (Urban and Rural) (n=48,476)		Urban (n=11,821)		Rural (n=36,655)	
	n	% (95% CI)	n	% (95% CI)	n	% (95% CI)
All	1,272	2.6 (2.5–2.8)	412	3.5 (3.2–3.8)	860	2.3 (2.2–2.5)
Province						
TORBA (n=8,569)	257	3.0 (2.7–3.4)	-	-	257	3.0 (2.7–3.4)
SANMA (n=39,907)	1,015	2.5 (2.4–2.7)	412	3.5 (3.2–3.8)	603	2.1 (2.0–2.3)
Sex						
Male (n=24,808)	681	2.7 (2.5–3.0)	212	3.5 (3.1–4.0)	469	2.5 (2.3–2.7)
Female (n=23,668)	591	2.5 (2.3–2.7)	200	3.4 (3.0–3.9)	391	2.2 (2.0–2.4)
Age group						
5-17 years (n=17,160)	263	1.5 (1.4–1.7)	81	2.1 (1.7–2.6)	182	1.4 (1.2–1.6)
18-35 years (n=16,954)	251	1.5 (1.3–1.7)	72	1.7 (1.3–2.1)	179	1.4 (1.2–1.6)
36-49 years (n=7,491)	195	2.6 (2.3–3.0)	79	4.0 (3.2–5.0)	116	2.1 (1.8–2.5)
50+ years (n=6,871)	563	8.2 (7.6–8.9)	180	10.6 (9.2–12.1)	383	7.4 (6.7–8.2)
18+ years (n=31,316)	1,009	3.2 (3.0–3.4)	331	4.2 (3.7–4.6)	678	2.9 (2.7–3.1)
Limitation Type¤						
Seeing	381	0.8 (0.7–0.9)	127	1.1 (0.9–1.3)	254	0.7 (0.6–0.8)
Hearing	320	0.7 (0.6–0.7)	106	0.9 (0.7–1.1)	213	0.6 (0.5–0.7)
Mobility	542	1.1 (1.0–1.2)	162	1.4 (1.2–1.6)	380	1.0 (0.9–1.1)
Memory	240	0.5 (0.4–0.6)	81	0.7 (0.6–0.9)	159	0.4 (0.4–0.5)
Self-Care	211	0.4 (0.4–0.5)	60	0.5 (0.4–0.7)	151	0.4 (0.4–0.5)
Communication	204	0.4 (0.4–0.5)	60	0.5 (0.4–0.7)	144	0.4 (0.3–0.5)

¤ Not mutually exclusive.

The most common functional limitations were with mobility, seeing and hearing. Not included in the disability prevalence estimate, the prevalence of depression (2.4%, 2.2–2.6) and anxiety (1.7%, 1.6–1.9) were high compared to other types of limitation, and the latter statistically more common in women [Web Table 1]. Over one third (37.4%, 36.3–38.5) of adults aged 50 years or older reported some difficulty seeing, whilst 23.7% (22.7–24.7) reported some difficulty with mobility. The discrimination people with a disability face in society emerged clearly from the qualitative data. For example, one participant explained how her status in the community reduced when she became disabled.

*“To me, they don't respect us, I mean who are we? We are useless to the community. What purpose do we serve in this society? I am talking straight and am being bold about it because it is the truth. People do not respect people like us”* (Woman, 61-year-old, disability, experiences incontinence, urban).

In some smaller communities, complete matching of cases and controls was not achieved, meaning that 814 people with disabilities and 702 people without disabilities were enrolled into the nested case-control study. No household-level disparities in access to WASH facilities (see Table 1 for definitions) between households with or without at least one member with a disability were observed (data not shown). Household-level access to WASH facilities were compared between rural and urban dwellings (Table 4). 99% of urban households and 89% of rural households had access to an improved drinking water source. Compared with urban households, rural households were more likely to use surface water (SES-adjusted odds ratio [adjOR] 7.6, 95% Confidence Interval 1.8–32.0). Rural households were also more likely to travel up to, or over, 30 minutes (return) to access water (adjOR 8.1, 5.5–11.8 and adjOR 19.7, 7.3–53.0, respectively). Rural households were less likely to use an improved sanitation facility compared with urban households (adjOR 0.5, 0.4–0.8), or to share their facility (adjOR 0.6, 0.4–0.7). One participant, who is unable to sit up out of bed unaided, explained how her four-year-old child supports her by collecting water, cleaning her bucket latrine and preparing food. Due to the full-time nature of care required, she had to withdraw her son from pre-school.

“..my youngest child was at kindy and I was paying his fees but now I've ruined his education, because he's my right hand. And because of my leg… and to urinate and defecate and go to the toilet, so I keep him at home. When I was ok, I had many friends and family… I have a stepmother… but when I fell down, the person who helps me is my youngest child *(Woman, 42 years, disability–walking, self-care, incontinence and MHM, urban)*

**Table 4:** Household level access to drinking water and sanitation.

	All Households (n=1516)		Urban Households (n=348)		Rural Households (n= 1,168)		SES adjusted Odds Ratio (95% CI)
	n	%	n	%	n	%	
Water source							
Improved	1387	91%	345	99%	1042	89%	0.2 (0.1–0.5)ǂ
Unimproved	129	9%	3	1%	126	11%	Reference
Drinking Water Ladder Level							
Basic	1303	86%	340	98%	963	82%	Reference
Limited	84	6%	5	1%	79	7%	2.6(1.0–6.9)
Unimproved	33	2%	1	1%	32	3%	5.4 (0.7–42.5)
Surface Water	96	6%	2	1%	94	8%	7.6 (1.8–32.0)ǂ
Water Source Location							
On premises/ piped into dwelling	207	14%	145	42%	62	5%	Reference
Less than 30 Minutes round trip	1174	77%	198	57%	976	84%	8.1 (5.5–11.8) ǂǂ
More than 30 minutes round trip	135	9%	5	1%	130	11%	19.7 (7.3–53.0) ǂǂ
Sufficiency of water supply¥							
Always sufficient	651	43%	156	45%	495	42%	Reference
Sometimes sufficient	619	41%	147	42%	472	40%	0.9 (0.7–1.2)
Never sufficient	229	15%	39	11%	190	16%	1.1 (0.7–1.8)
Don't know	17	1%	6	2%	11	1%	0.5 (0.2–1.8)
Sanitation Facility							
Improved	1,144	75%	310	89%	834	71%	0.5 (0.4–0.8)ǂ
Unimproved	372	25%	38	11%	334	29%	Reference
Facility is shared§	546	38%	161	48%	385	34%	0.6 (0.4–0.7)ǂǂ
Sanitation Ladder Level							
Basic	722	48%	168	48%	554	47%	Reference
Limited	422	28%	142	41%	280	24%	0.5 (0.4–0.7)ǂǂ
Unimproved	311	21%	28	8%	283	24%	1.7 (1.0–2.6)ǂ
Open Defecation	61	4%	10	3%	51	4%	0.8 (0.3–1.7)

¥ In the last month.

§ Excludes households that practice open defecation.

ǂǂ p<0.001 or

ǂ p<0.05 binary or multinomial multivariate logistic regression.

Within their households, people with disabilities consistently reported increased barriers to adequate WASH compared with people without disabilities, in both rural and urban locations (Table 5). During an in-depth interview, one person with a disability reported that she became disabled when she slipped and fell on the way to her toilet, underlining the serious consequences of inaccessible facilities.

**Table 5:** Intra Household WASH characteristics.

	All Households					Urban Households					Rural Households				
	People with disabilities (n=814)		People without disabilities (n=702)		Adj. Odds Ratio (95% CI)⁎⁎	People with disabilities (n=190)		People without disabilities (n=158)		Adj. Odds Ratio (95% CI)⁎⁎	People with disabilities (n=624)		People without disabilities (n=544)		Adj. Odds Ratio (95% CI)⁎⁎
	n	%	n	%		n	%	n	%		n	%	n	%	
Access water at home when need it	734	90%	697	99%	0.1 (0.1 – 0.2) ǂǂ	165	87%	157	99%	0.1 (0.1 – 0.3)ǂ	569	91%	540	99%	0.1 (0.1 – 0.2)ǂǂ
Collect water themselves (all)§	502	66%	589	93%	0.2 (0.1 – 0.2)ǂǂ	94	67%	97	94%	0.2 (0.1 – 0.2)ǂǂ	408	66%	492	93%	0.1 (0.1 – 0.3)ǂǂ
Feel safe when collecting water∝	451	84%	602	95%	0.3 (0.2 – 0.4) ǂǂ	112	90%	125	95%	0.5 (0.2 – 1.3)	339	82%	477	95%	0.2 (0.1 – 0.4)ǂǂ
Use the same facility as other members of household	694	86%	684	98%	0.1 (0.1 – 0.2)ǂǂ	166	88%	152	96%	0.3 (0.1 – 0.7)ǂ	528	85%	553	98%	0.1 (0.1 – 0.2) ǂǂ
Materials are available to clean self after using the toilet	599	75%	521	74%	1.0 (0.8 – 1.2)	160	85%	134	85%	1.0 (0.5 – 1.8)	439	71%	388	71%	1.0 (0.7 – 1.2)
Need assistance to use toilet	307	38%	127	18%	2.9 (2.2 – 3.7)ǂǂ	72	38%	31	20%	2.6 (1.6 – 4.4) ǂǂ	235	38%	96	18%	3.0 (2.2 – 3.9)ǂǂ
Difficult to use toilet without coming into contact with faeces or urine	261	32%	99	14%	3.0 (2.3 – 3.9)ǂǂ	54	29%	25	16%	2.1 (1.2 – 3.6)ǂǂ	207	33%	74	14%	3.3 (2.5 – 4.5) ǂǂ
Able to use toilet as frequently as desire	713	88%	688	98%	0.1 (0.1 – 0.2)ǂǂ	168	89%	156	99%	0.1 (0.1 – 0.5)ǂǂ	545	88%	533	98%	0.1 (0.1 – 0.3)ǂǂ

⁎⁎ Age, Sex, Location, SES adjusted

§ Excludes 59 cases (49 urban) and 68 controls (55 urban) whose drinking water supply is piped directly into the dwelling

∝ amongst those who collect water themselves

ǂǂ p<0.001 or

ǂ p<0.05 binary multivariable logistic regression

People with disabilities were statistically more likely (all p<0.001) to experience barriers in each of eight indicators of intra-household WASH access except having materials available to clean themselves after using the toilet (p≥0.05). These associations held when stratified by rural or urban location in all instances except the collection of water in Luganville. Multivariable regression models were built to explore predictors among people with disabilities for three water (Web Table 2) and three sanitation (Web Table 3) indicators. Older people with disabilities were more likely not to collect water themselves (adjOR 2.0, 1.2–3.3), and less likely to need assistance (adjOR 0.5, 0.3–0.7) or report it being difficult to use the toilet hygienically (adjOR 0.5, 0.3–0.7) compared with younger people with disabilities. Women with disabilities were twice as likely (adjOR 1.6, 1.2–2.2) as men with disabilities to report needing assistance, but there were no other statistical associations by sex (p≥0.05). Mobility limitations were associated with each of the six indicators, whilst self-care limitations were associated with each indicator except feeling safe when collecting water.

A caregiver explained that the route to the family toilet is unsafe, so the person he supports with a disability does not attempt to reach it independently. Without any incontinence products (e.g. handheld urinal), or assistive devices, such as a wheelchair, the young man stays where he is and soils himself.

*“It's risky for him to go to the toilet because he might fall in so he just goes out like this and sometimes he just makes a mess of himself and sits quietly until he gets cleaned up and sometimes when waking up in the morning he doesn't have control and messes himself”* (Proxy in-depth interview, man, 18 years, multiple functional limitations, experiences incontinence, rural).

**Fig. 1:**
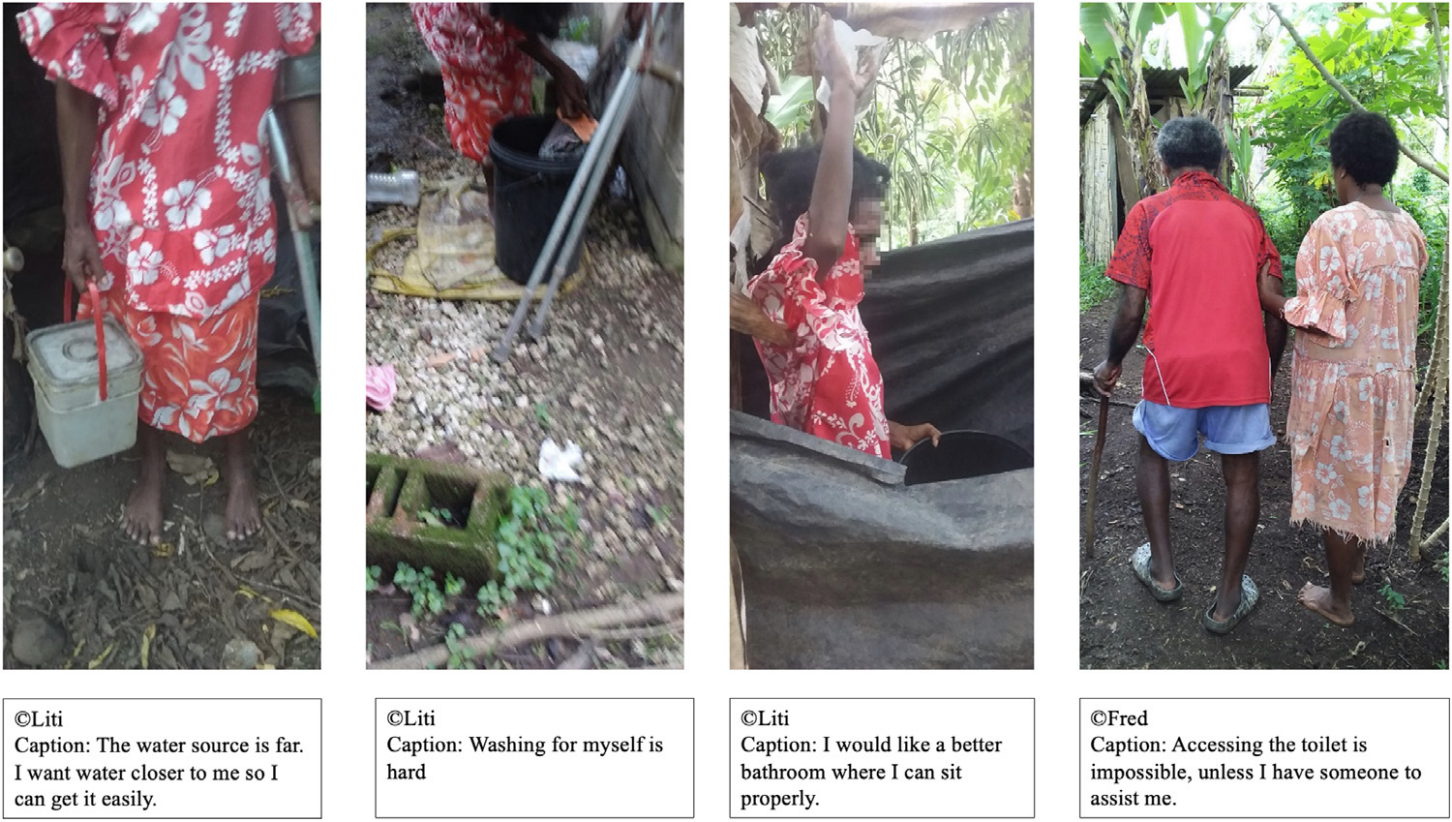
Liti and fred photovoice images.

In Vanuatu, sociocultural beliefs around menstruation dictate that women must collect their own water for washing their menstrual material and bathing, wash their own menstrual product independently and use separate bathing shelters and latrines when menstruating. Fig. 1 includes three PhotoVoice outputs by Liti, a female participant with a mobility limitation, highlighting the layered difficulties she experiences when menstruating: the implications of needing to travel to the water source, the inaccessible facilities and the lack of support. Liti described extreme back pain made worse through the exertion of collecting and using water, in particular during menstruation. Fig. 1 also shows Fred, an older male participant who experiences incontinence, being assisted to the toilet by his wife. The photo depicts the reliance on informal caregivers, and how additional requirements for WASH for people who menstruate and/or experience incontinence are often unmet.

Table 6 presents the proportion of people with and without disabilities classified as “suffering”, “struggling” or “thriving” using the Cantril Self-Anchoring Striving scale. People with disabilities were over ten times more likely to be “suffering” (adjOR 10.5, 6.4–17.3) and three times more likely to be “struggling” (adjOR 3.0, 2.3–3.9) than people without disabilities. Compared to men without disabilities, women without disabilities were half as likely to be “thriving” (adjOR 0.5, 0.4–0.8) and men and women with disabilities were five times less likely to be “thriving” (women: adjOR 0.2, 0.2–0.4; men: adjOR 0.2, 0.1–0.2). In an in-depth interview, a participant described how she feels neglected because of her perceived declining value in the family.

*“I'm all alone [….] I'll just be here… if I'm hungry I'll go look for them [family], but they don't care for me”* (Woman, 74-year-old, disability, experiences incontinence, rural).

**Table 6:** (age 16+).

	People with disabilities (n=641)		People without disabilities (n=540)		Age, Sex, Location, SES adjusted Odds Ratio (95% CI)
	n	%	n	%	
**Wellbeing**					
Suffer	126	20%	24	4%	10.5 (6.4–17.3)ǂǂ
Struggle	385	60%	262	49%	3.0 (2.3–3.9)ǂǂ
Thrive	130	20%	254	47%	Reference

	Thriving (n = 384)				Location, SES adjusted Odds Ratio (95% CI)
	**n**		**%**		

**Sex and Disability group**					
Male–no disability	143		37%		Reference
Female–no disability	111		20%		0.5 (0.4–0.8) ǂǂ
Male–disability	77		29%		0.2 (0.2–0.4)ǂ
Female - disability	53		14%		0.2 (0.1–0.2) ǂǂ

ǂǂ p<0.001 or

ǂ p<0.05 multinomial multivariable logistic regression.

Results from the key informant interviews with policy makers identified a number of areas for strengthening the WASH policy context in Vanuatu to better support people with disabilities. Responsibility for WASH spans several ministries, with civil society organisations (CSOs) invited to participate in policy development. However, CSO key informants expressed exclusion from policy development/ co-design once they had provided the requested contributions and described the tension of competing objectives among different stakeholders (for example gender, disability or education activists). In addition, chronic understaffing in the ministries was found to be exacerbated by frequent humanitarian response demands, such as the recent Ambae emergency response in SANMA. The appointment of provincial water officers in TORBA and SANMA, and the reference to inclusiveness as a guiding principle in draft government sanitation guidelines were seen as strengths. However, the limited visibility of hygiene across WASH policies was described as a key challenge by a key informant.

*“… hygiene is a topic that is quite often overlooked and not taken seriously. And it takes sometimes some champions […] to remind us that it's very important and that we can't ignore the H in the WASH”* (Government official, Vanuatu)*.*

## 4 Discussion

This mixed methods study provides an unparalleled breadth and depth of evidence on the WASH experiences of people with disabilities in TORBA and SANMA, the two northern-most provinces of Vanuatu. This is the first publication from a series that will explore the findings in more detail.

The all-age disability prevalence estimate (2.6%) is higher than the 2009 Vanuatu Census (0.8%) but not directly comparable as the latter modified the WGSS response options to exclude the highest category “unable to do” [45]. The estimate is considerably lower than the 15% estimated by the 2011 World Report on Disability, but notably similar to other findings using the same approach in two other Pacific Island States, Samoa and Kiribati [46], [47], [48]. Beyond differences in measurement approaches compared to the World Report on Disability, this may in part also be due to the population age structure, which is relatively young (42% under 18 versus 32% in the WHO Standard Population [49]). Given the association between disability and ageing, a younger population are likely to have a lower all-age estimate of disability. There may also be cultural reasons that contribute to these relatively low estimates in the Pacific compared to other settings. For example, small island communities across the Pacific have been shown to be resilient and adaptable in the face of colonial occupation, successive natural disasters and, more recently, climate change [50]. Interpretation of the response categories of the WGSS in the Pacific, and their comparability to other settings, warrants further exploration. Additionally, 22.2% of participants across the two provinces reported “some” or greater difficulty in at least one functional domain. This high proportion of the population reporting a degree of functional limitation reinforces the imperative for disability-inclusive WASH, irrespective of the comparatively low disability prevalence estimate using standard definitions. A review of the analytic properties of the WGSS standard definition in the Pacific context should also be completed, in light of Pacific findings.

Disability prevalence was similar by sex but increased with age, as is commonly seen [51], [52], [53]. This latter finding is particularly important given that functional decline related to ageing is often perceived culturally as distinct from disability, when in fact the restrictions on participation and implications on quality of life are interconnected [54]. Particularly given the current and projected acceleration in population ageing across the Pacific, investment in the physical, social and economic determinants of healthy ageing across the life-course are imperative [55,56].

The vast majority of rural and urban households included in the case-control study had access to an improved water source, but over half reported insufficiency of the water supply in the last month. Qualitative interviews highlighted the profound impacts of not having accessible WASH facilities on people's lives. Particularly in the context of the ongoing COVID-19 pandemic, availability of a reliable supply of safely managed and affordable drinking water is a critical public health requirement, and a mandatory foundational step in meeting all population WASH requirements, including those of people with disabilities, people who experience incontinence and girls and women who menstruate [57].

Access to an improved sanitation facility was not universal (89% Luganville, 71% rural settings) across the two provinces, with facilities more likely to be shared in Luganville and people with disabilities in both rural and urban environments significantly more likely to require assistance, come into contact with waste matter, and have to self-limit their use of facilities than people without disabilities. This increases the risk of chronic diseases related to dehydration and faecal contamination, further urinary and bowel impairment, hygiene-related stigma, and potentially increases risk of COVID-19 transmission [58,59]. The implications of barriers to WASH and participation more generally are reflected in the self-rated wellbeing scale and IDIs, highlighting how gender and disability intersect to decrease wellbeing in the two provinces.

The study identified numerous tangible recommendations for the development of holistic, inclusive programmes and policies to meet population needs, including meeting the additional requirements of specific groups such as women and girls who menstruate, and people who experience incontinence. These included emphasis on both strengthening the consistency of household water supplies and prioritising self-supply initiatives; on destigmatisation activities; and on the establishment of local supply chains for incontinence and menstrual products. At a policy level, the study highlighted the need for a single and fully inclusive WASH policy across the numerous involved ministries. More broadly, since the results have been disseminated in Vanuatu, local stakeholders perceive an increased commitment to disability-inclusive disaster risk reduction strategies and responses by other agencies and response systems, through heightened awareness of barriers and challenges experienced by marginalised groups.

Finally, in response to Cyclone Harold, the LDK team were also able to use the study data to prioritise their responses, using disaggregated sub-provincial data and contact details of participants identified to have additional requirements, and selecting disability-inclusive distribution sites. Activities included building back accessible latrines for 100 households of people with disabilities, providing menstrual hygiene and incontinence management materials for 100 people with disabilities, and prioritising rainwater system repair. In addition, survey interviewers were re-engaged within household registration teams, capitalising on their skills in collecting accurate disability population data in a sensitive manner.

The Vanuatu Water, Women and Disability study was the first of its kind, and encountered several operational and logistical challenges. Menstruation and incontinence are sensitive topics, with some terms and phrases having no direct translation in Bislama. Despite in-depth training and a comprehensive field manual, some data collectors struggled with interpretation of key themes and internalised stigma/taboo, which required re-training and reinforcement of study principles. However, the study also has several core strengths. The combination of mixed methods provided a rich and comprehensive baseline assessment for the LDK project and for SANMA and TORBA provinces as a whole. The total population listing and disability prevalence survey provided baseline data and gave the overall view of key trends, while the qualitative research delved into reasons for these trends and identified areas of intervention. While it is hoped that Vanuatu can succeed in continuing to prevent COVID-19 infection across the country, the study findings also provide the necessary data on safely managed water, sanitation and hygiene services to strengthen risk mitigation and prevention of infection, particularly for vulnerable groups.

## 5 Conclusion

This mixed-methods study has provided rich, valuable data on disability and WASH in Vanuatu, including the additional, largely unmet requirements of women and girls who menstruate and people with incontinence. It highlights the continued challenges experienced by these populations in accessing safe, affordable and appropriate drinking water, sanitation and hygiene, and the key recommendations to development actors to overcome these. The findings have been used to develop inclusive programmes and responses to Tropical Cyclone Harold, and will be used to support continued COVID-19 preparedness and response.

## Author contributions

IZM: Literature Search, Study Conceptualization, Study Design, Data Collection Oversight, Verification of underlying data, Data Analysis, Data Interpretation, Manuscript Writing

SB: Study Design, Data Interpretation, Manuscript Review

LB: Study Design, Data Collection Oversight, Data Interpretation, Manuscript Review

JI: Data Collection, Data Interpretation, Manuscript Review

MJK: Data Analysis Review, Data Interpretation, Manuscript Review

CM: Data Collection Oversight, Data Interpretation, Manuscript Review

RP: Study Design, Data Collection Oversight, Data Interpretation, Manuscript Review

JS: Data Collection, Data Interpretation, Manuscript Review

PS: Literature Search, Study Design, Data Collection Oversight, Data Interpretation, Manuscript Review

JT: Study Design, Data Collection Oversight, Data Interpretation, Manuscript Review

JW: Literature Search, Study Conceptualization, Study Design, Data Collection Oversight, Verification of underlying data, Data Analysis, Data Interpretation, Manuscript Writing

IZM, PS, JT and JW had full access to the full data in the study, and accept responsibility to submit for publication.

## Declaration of Competing Interest

Dr. Mactaggart, Ms. Wilber and Mr. Sheppard reported grants from World Vision Vanuatu, during the conduct of the study; Ms. Baker, Mr. Bambery, Ms. Iakavai, Dr. Kim, Ms. Morrison, Ms. Poilapa, Ms. Shem and Mr. Tanguay have nothing to disclose.

## Appendix Supplementary materials

Image, application 1
