# Supplementary material for: Water, women and disability: Using mixed-methods to support inclusive WASH programme design in Vanuatu
Source: Lancet Reg Health West Pac. 2021 Mar 2;8:100109. doi: 10.1016/j.lanwpc.2021.100109 (PMC8315363; doi:10.1016/j.lanwpc.2021.100109)
Supplement: Supplementary file 1 [file mmc1.docx]

| Web Table: Prevalence by functional domain | | | | | | | | | | | | | | |
| --- | --- | --- | --- | --- | --- | --- | --- | --- | --- | --- | --- | --- | --- | --- |
|  | All ages (n=48,476) | | Male (n=24,808) | | Female (n=23,668) | | 5-17 (n=17,322) | | 18-35 (n=16,978) | | 36-49 (n=7,504) | | 50+ (n=8,955) | |
|  | n | % (95% CI) | n | % (95% CI) | n | % (95% CI) | n | % (95% CI) | n | % (95% CI) | n | % (95% CI) | n | % (95% CI) |
| ***Seeing*** |  |  |  |  |  |  |  |  |  |  |  |  |  |  |
| A lot/ Can’t | 381 | 0.8 (0.7 – 0.9) | 204 | 0.8 (0.7 – 0.9) | 177 | 0.7 (0.6 – 0.9) | 48 | 0.3 (0.2 – 0.4) | 65 | 0.4 (0.3 – 0.5) | 58 | 0.8 (0.6 – 1.0)^¥^ | 210 | 3.1 (2.7 – 3.5) ^¥^ |
| Some | 4,618 | 9.5 (9.3 – 9.8) | 2,325 | 9.4 (9.0 – 9.7) | 2,293 | 9.7 (9.3 – 10.1) | 152 | 0.9 (0.8 – 1.0) | 622 | 3.7 (3.4 – 4.0)^¥^ | 1,275 | 17.0 (16.2 – 17.9)^¥^ | 2,569 | 37.4 (36.3 – 38.5) ^¥^ |
| ***Hearing*** |  |  |  |  |  |  |  |  |  |  |  |  |  |  |
| A lot/ Can’t | 320 | 0.7 (0.6 – 0.7) | 170 | 0.7 (0.6 – 0.8) | 150 | 0.6 (0.5 – 0.7) | 86 | 0.5 (0.4 – 0.6) | 54 | 0.3 (0.2 – 0.4) | 44 | 0.6 (0.4 – 0.8) | 136 | 2.0 (1.7 – 2.3) ^¥^ |
| Some | 2,533 | 5.2 (5.0 – 5.4) | 1,347 | 5.4 (5.2 – 5.7) | 1,186 | 5.0 (4.7 – 5.3) | 594 | 3.5 (3.2 – 3.7) | 465 | 2.7 (2.5 – 3.0) | 403 | 5.4 (4.9 – 5.9)^¥^ | 1,071 | 15.6 (14.7 – 16.5) ^¥^ |
| ***Mobility*** |  |  |  |  |  |  |  |  |  |  |  |  |  |  |
| A lot/ Can’t | 542 | 1.1 (1.0 – 1.2) | 286 | 1.2 (1.0 – 1.3) | 256 | 1.1 (1.0 – 1.2) | 85 | 0.5 (0.4 – 0.6) | 71 | 0.4 (0.3 – 0.5) | 77 | 1.0 (0.8 – 1.3)^¥^ | 309 | 4.5 (4.0 – 5.0) |
| Some | 3,332 | 6.9 (6.7 – 7.1) | 1,561 | 6.3 (6.0 – 6.6) | 1,771 | 7.5 (7.2 – 7.8)^ǂ^ | 219 | 1.3 (1.1 – 1.5) | 703 | 4.1 (3.9 – 4.5)^¥^ | 782 | 10.4 (9.8 – 11.2)^¥^ | 1,628 | 23.7 (22.7 – 24.7) ^¥^ |
| ***Memory*** |  |  |  |  |  |  |  |  |  |  |  |  |  |  |
| A lot/ Can’t | 240 | 0.5 (0.4 – 0.6) | 138 | 0.6 (0.5 – 0.7) | 102 | 0.4 (0.4 – 0.5) | 71 | 0.4 (0.3 – 0.5) | 78 | 0.5 (0.4 – 0.6) | 24 | 0.3 (0.2 – 0.5) | 67 | 1.0 (0.8 – 1.2) ^¥^ |
| Some | 3,198 | 6.7 (6.4 – 6.8) | 1,651 | 6.7 (6.4 – 7.0) | 1,547 | 6.5 (6.2 – 6.9) | 482 | 2.8 (2.6 – 3.1) | 954 | 5.6 (5.3 – 6.0)^¥^ | 691 | 9.2 (8.6 – 9.9) ^¥^ | 1,071 | 15.6 (14.7 – 16.5) ^¥^ |
| ***Self-Care*** |  |  |  |  |  |  |  |  |  |  |  |  |  |  |
| A lot/ Can’t | 211 | 0.4 (0.4 – 0.5) | 120 | 0.5 (0.4 – 0.6) | 91 | 0.4 (0.3 – 0.5) | 64 | 0.4 (0.3 – 0.5) | 36 | 0.2 (0.2 – 0.3) | 15 | 0.2 (0.1 – 0.3) | 96 | 1.4 (1.1 – 1.7) ^¥^ |
| Some | 321 | 0.7 (0.5 – 0.7) | 173 | 0.7 (0.6 – 0.8) | 148 | 0.6 (0.5 – 0.7) | 81 | 0.5 (0.4 – 0.6) | 72 | 0.4 (0.3 – 0.5) | 40 | 0.5 (0.4 – 0.7) | 128 | 1.9 (1.6 – 2.2) ^¥^ |
| ***Comm.*** |  |  |  |  |  |  |  |  |  |  |  |  |  |  |
| A lot/ Can’t | 204 | 0.4 (0.4 – 0.5) | 105 | 0.4 (0.3 – 0.5) | 99 | 0.4 (0.3 – 0.5) | 74 | 0.4 (0.3 – 0.5) | 69 | 0.4 (0.3 – 0.5) | 29 | 0.4 (0.3 – 0.6) | 32 | 0.5 (0.3 – 0.7) |
| Some | 477 | 1.0 (0.9 – 1.1) | 232 | 0.9 (0.8 – 1.1) | 245 | 1.0 (0.9 – 1.2) | 177 | 1.0 (0.9 – 1.2) | 147 | 0.9 (0.7 – 1.0) | 56 | 0.7 (0.6 - 1.0) | 97 | 1.4 (1.2 – 1.7) ^¥^ |
| *Affect domains: not included in prevalence estimate NB adult (18+) only* | | | | | | | | | | | | | | |
| ***Anxiety*** | 388 | 1.7 (1.6 – 1.9) | 149 | 1.4 (1.2 – 1.7) | 239 | 2.0 (1.8 – 2.3)^ǂ^ | - | - | 122 | 1.1 (0.9 – 1.3) | 119 | 2.1 (1.8 – 2.6)^¥^ | 147 | 2.7 (2.3 – 3.2) |
| ***Depression*** | 534 | 2.4 (2.2 - 2.6) | 246 | 2.3 (2.1 – 2.6) | 288 | 2.4 (2.1 – 2.7) | - | - | 253 | 2.2 (1.0 – 2.5) | 148 | 2.6 (2.3 – 3.1) | 133 | 2.5 (2.1 – 2.9) |
| ^ǂ^Statistically different by sex  ^¥^Statistically different compared to age group below | | | | | | | | | | | | | | |

| Web Table 2 Factors associated with collecting and using water amongst people with disabilities (n=642) | | | | | | | | | | |
| --- | --- | --- | --- | --- | --- | --- | --- | --- | --- | --- |
|  | Don’t Collect water themselves | | | | Don’t Feel Safe collecting water^§^ | | | Can’t access water at home when need it | | |
|  | n | % | | Age, Sex, Location, SES adj. Odds Ratio (95% CI) | n | % | Age, Sex, Location, SES adj. Odds Ratio (95% CI) | n | % | Age, Sex, Location, SES adj. Odds Ratio (95% CI) |
| **Age Group** |  | |  |  |  |  |  |  |  |  |
| 5 – 17 | 54 | | 30% | Reference | 20 | 15% | Reference | 24 | 12% | Reference |
| 18 – 49 | 67 | | 24% | 1.0 (0.6 – 1.7) | 39 | 18% | 1.2 (0.6 – 2.2) | 19 | 6% | 0.7 (0.3 – 1.4) |
| 50+ | 133 | | 44% | 2.0 (1.2 – 3.3) ^ǂ^ | 27 | 15% | 0.9 (0.4 – 1.8) | 36 | 11% | 1.0 (0.5 – 2.0) |
| **Sex** |  | |  |  |  |  |  |  |  |  |
| Male | 140 | | 36% | Reference | 40 | 15% | Reference | 47 | 11% | Reference |
| Female | 114 | | 31% | 0.8 (0.5 – 1.1) | 46 | 17% | - 1. (0.7 – 1.8) | 32 | 8% | 0.7 (0.4 – 1.2) |
| **Location** |  | |  |  |  |  |  |  |  |  |
| Rural | 207 | | 34% | 1.0 (0.6 – 1.6) | 74 | 18% | 0.7 (0.4 – 1.6) | 55 | 9% | 2.0 (1.0 – 4.1) |
| Urban | 47 | | 34% | Reference | 12 | 10% | Reference | 24 | 13% | Reference |
| **Limitation type** |  | |  |  |  |  |  |  |  |  |
| Seeing | 58 | | 28% | 0.9 (0.6 – 1.5) | 21 | 13% | 1.0 (0.5 – 1.8) | 14 | 6% | 0.8 (0.4 – 1.6) |
| Hearing | 27 | | 15% | 0.4 (0.3 – 0.7)^ǂ^ | 19 | 12% | 0.8 (0.4 – 1.5) | 9 | 5% | 0.7 (0.3 -1.5) |
| Mobility | 179 | | 52% | 3.0 (2.0 – 4.6) ^ǂǂ^ | 42 | 24% | 2.4 (1.3 – 4.4) ^ǂ^ | 64 | 17% | 4.0 (2.1 – 7.7) ^ǂǂ^ |
| Memory | 53 | | 35% | 1.1 (0.7 – 1.9) | 21 | 20% | 1.6 (0.8 – 3.1) | 25 | 15% | 1.1 (0.5 – 2.2) |
| Self-Care | 107 | | 76% | 10.0 (6.0 – 16.8) ^ǂǂ^ | 8 | 21% | 1.0 (0.4 – 2.6) | 46 | 30% | 4.9 (2.8 – 8.6)^ǂǂ^ |
| Communication | 39 | | 28% | 0.6 (0.3 – 1.0) | 16 | 16% | 0.8 (0.4 – 1.6) | 25 | 17% | 2.0 (1.0 – 4.1) |
| ^ǂǂ^ p<0.001 or ^ǂ^p<0.05 binary or multinomialmultivariable logistic regression  ^§^Amongst people with disabilities who collect water themselves  Notes: All variables in table included in one multivariable model. Functional limitation variables are binary (does have vs does not have) and are not mutually exclusive, as people may have more than one limitation | | | | | | | | | | |

| Web Table 3 Factors associated with accessing the toilet amongst people with disabilities (n=809) | | | | | | | | | | |
| --- | --- | --- | --- | --- | --- | --- | --- | --- | --- | --- |
|  | Need assistance | | | | Difficult to use without coming into contact with faeces or urine | | | Not able to use as frequently as desire | | |
|  | n | % | | Age, Sex, Location, SES adjusted Odds Ratio (95% CI) | n | % | Age, Sex, Location, SES adjusted Odds Ratio (95% CI) | n | % | Age, Sex, Location, SES adjusted Odds Ratio (95% CI) |
| **Age Group (years)** |  | |  |  |  |  |  |  |  |  |
| 5 – 17 | 109 | | 56% | Reference | 91 | 46% | Reference | 33 | 17% | Reference |
| 18 – 49 | 81 | | 27% | 0.3 (0.2 – 0.5)^ǂǂ^ | 73 | 25% | 0.4 (0.3 – 0.6)^ǂǂ^ | 27 | 9% | 0.7 (0.4 – 1.4) |
| 50+ | 117 | | 37% | 0.5 (0.3 – 0.7)^ǂǂ^ | 97 | 31% | 0.5 (0.3 – 0.7)^ǂ^ | 35 | 11% | 0.8 (0.4 – 1.5) |
| **Sex** |  | |  |  |  |  |  |  |  |  |
| Male | 143 | | 34% | Reference | 128 | 31% | Reference | 50 | 12% | Reference |
| Female | 164 | | 42% | 1.6 (1.2 – 2.2)^ǂǂ^ | 133 | 34% | 1.3 (0.9 – 1.8) | 47 | 12% | 1.1 (0.7 – 1.8) |
| **Location** |  | |  |  |  |  |  |  |  |  |
| Rural | 235 | | 38% | 1.0 (0.7 – 1.5) | 207 | 33% | 0.8 (0.5 – 1.2) | 74 | 12% | 0.8 (0.4 – 1.6) |
| Urban | 72 | | 38% | Reference | 54 | 29% | Reference | 21 | 11% | Reference |
| **Limitation type** |  | |  |  |  |  |  |  |  |  |
| Seeing | 70 | | 31% | 0.8 (0.6 – 1.3) | 60 | 27% | 1.1 (0.7 – 1.6) | 18 | 8% | 0.8 (0.5 – 1.5) |
| Hearing | 52 | | 28% | 0.6 (0.4 – 1.0) | 46 | 25% | 0.8 (0.5 – 1.3) | 13 | 7% | 0.7 (0.4 – 1.4) |
| Mobility | 170 | | 47% | 1.5 (1.1 – 2.4) ^ǂ^ | 156 | 43% | 2.2 (1.5 – 3.3) ^ǂǂ^ | 69 | 19% | 2.5 (1.4 – 4.3) ^ǂǂ^ |
| Memory | 89 | | 53% | 1.7 (1.1 – 2.6) ^ǂ^ | 73 | 44% | 1.5 (1.0 – 2.3) | 37 | 22% | 1.8 (1.0 – 3.3) ^ǂ^ |
| Self-Care | 109 | | 72% | 4.1 (2.7 – 6.3)^ǂǂ^ | 96 | 63% | 3.4 (2.2 – 5.1) ^ǂǂ^ | 56 | 37% | 5.7 (3.4 – 9.5)^ǂǂ^ |
| Communication | 71 | | 48% | 1.1 (0.7 – 1.8) | 63 | 63% | 1.3 (0.8 – 2.0) | 33 | 22% | 1.6 (0.8 – 2.9) |
| ^ǂǂ^ p<0.001 or ^ǂǂ^ p<0.05 multinomial multivariable logistic regression  Notes: All variables in table included in one multivariable model. Functional limitation variables are binary (does have vs does not have) and are not mutually exclusive, as people may have more than one limitation | | | | | | | | | | |
